# Outcomes of Interventions for Angiosarcoma

**DOI:** 10.3389/fsurg.2022.819099

**Published:** 2022-04-11

**Authors:** Qi Yan, Roman A. Fernandez, Maryam Elmi, Jonathan Gelfond, Mark G. Davies

**Affiliations:** ^1^Department of Surgery, Long School of Medicine University of Texas Health at San Antonio, San Antonio, TX, United States; ^2^Department of Population Health Sciences, Long School of Medicine University of Texas Health at San Antonio, San Antonio, TX, United States; ^3^MD Anderson Cancer Center, Long School of Medicine University of Texas Health at San Antonio, San Antonio, TX, United States; ^4^Division of Vascular and Endovascular Surgery, Long School of Medicine University of Texas Health at San Antonio, San Antonio, TX, United States; ^5^South Texas Center for Vascular Care, South Texas Medical Center, San Antonio, TX, United States

**Keywords:** angiosarcoma, outcomes, survival, registry, intervention

## Abstract

**Objective:**

Angiosarcoma is a rare malignant vascular tumor, and the management and outcome of this disease are not well-described. The aim of this study was to report the incidence, patient demographics, and outcomes of angiosarcoma based on national data.

**Methods:**

Data on patients with angiosarcoma were obtained from the Surveillance, Epidemiology, and End Results (SEER) database. Inverse probability treatment weights (IPTW) were used to assess the survival benefit of operation with additional chemo or radiation therapy compared to operation alone. These variables were further compared against patients who did not receive an operation despite being initially offered one. Cox regression was used to assess survival. Statistical analyses were performed on RStudio.

**Results:**

For this study, 5,135 patients (46% men; median age 69, range 0–102) with angiosarcoma were identified in the SEER database between 1975 and 2016. The age-adjusted incidence rate was 1–4%. Patients were mostly non-Hispanic Caucasian (75.4%). The average tumor size was 4.7 cm, range (.1–98.9). Tumor grades were high at presentation (Grade III 17.2, Grade IV 19, and unknown 50.6%), but half were considered localized tumors. Most patients underwent an operation (66.1%). In 5.6% of patients, the operation was recommended but not performed. The overall 5-year survival was 26.7% (95% CI 25.4–28.1%). IPTW with adjusted Cox proportional hazard model demonstrated worse survival, showing that operation compared to no operation and operation with chemo/radiation compared to operation alone had worse survival between months 0 and 25 but had improved survival after month 25.

**Conclusions:**

The incidence of angiosarcoma is low and long-term survival is poor. Multimodal therapy in the form of neoadjuvant or adjuvant chemo/radiation therapy offers significant long-term survival benefits over operation alone.

## Introduction

Angiosarcoma is an aggressive neoplasm of the endothelial cells of blood or lymphatic vessels with a 5-year overall survival of 41–43%, affecting patients mostly in their sixth decade of life (1–3). It is a rare malignancy comprising only 4% of all soft tissue sarcomas (3, 4). These tumors can occur at any anatomical location but are most commonly found in the head, neck skin, and breast regions of the body (1). Risk factors for angiosarcoma include radiation, chronic lymphedema, radiotherapy, certain chemical exposure, and immunosuppression (5).

Due to the rarity of angiosarcoma, the sample sizes of large case series studies remain small with only around 200 patients reported (1, 2). Operation, radiotherapy, and chemotherapy are the main treatments for this disease, with the core treatment being surgical resection if possible (6). Specific treatment algorithms for angiosarcoma are currently lacking. However, retrospective studies on the outcomes of these treatment modalities have shown inconsistent results, possibly due to limited sample sizes (7–13). This study used the Surveillance, Epidemiology, and End Results (SEER) database, which allows for a unique examination of much larger cohorts than those currently reported in the literature. The reported 5-year overall survival rate of angiosarcoma was based on case series of single institutions in China and France (1, 2). On the other hand, US-based population studies on angiosarcoma using modern data are lacking. The SEER Program of registries within the National Cancer Institute has collected cancer case data from 19 US geographic areas that are thought to be representative of the demographics of the entire US population and thus allows for population-based cancer studies.

The primary aim of this study was to analyze 5-year overall survival of patients with primary angiosarcoma but without additional primary malignancy. The secondary aim of this study included the following: describe patient demographics, identify details of tumor characteristics of all patients with angiosarcoma using the national registry, explore the effect of primary site on the outcome, and identify factors associated with survival in primary angiosarcoma.

## Methods

### Study Design

This is a retrospective observational longitudinal survival study based on a prospectively maintained registry. All data were de-identified and thus does not require Institution Review Board approval or informed patient consent.

### Data Source

The SEER Program of the National Cancer Institute is considered an authoritative source for cancer statistics in the US (https://seer.cancer.gov/). SEER is supported by the Surveillance Research Program (SRP) in NCI's Division of Cancer Control and Population Sciences (DCCPS), and it collects data on cancer cases from various locations and sources throughout the US. For the current study, data from the SEER registry was accessed on October 7, 2019. The database SEER 18 Regs Custom Data (with additional treatment fields) on November 2018 Sub (1975–2016 varying) was used for patient demographics and survival analysis. SEER 18 covers approximately 28% of the US population (14). The database SEER 9 Regs Research Data on November 2018 Sub (1975–2016) was used for evaluation of the incidence.

### Search Strategy

Site and morphology based on the International Classification of Diseases for Oncology, Third Edition (ICD-O-3) of “9120/3: Hemangiosarcoma,” “9125/3: Epithelioid hemangiosarcoma,” and “9170/3: Lymphangiosarcoma” were used to retrieve all cases of angiosarcoma from 1975 to 2016.

### Inclusion Criteria

Patients with histologic confirmation of hemangiosarcoma, epithelioid hemangiosarcoma, and lymphangiosarcoma were included.

### Exclusion Criteria

Patients with missing age were excluded from the description of the patient cohort. Patients with another primary cancer either prior to or after having an angiosarcoma (including secondary angiosarcomas) were excluded from survival analysis. Death certificate or autopsy cases were excluded from the survival analysis. Tumor with the primary site of the prostate was excluded from survival analysis because there was only one case.

### Data Extraction

Demographic data obtained included age at diagnosis, race, and origin, gender, year of diagnosis, state-county, and the socioeconomic status (SES) index. The SES index in SEER is a time-dependent variable calculated using median household income, median house value, median rent, percent below 150% of the poverty line, education index, percent working class, and percent unemployed (15). Tumor data included primary site, grade, laterality, diagnostic confirmation, SEER historic stage, and tumor size. Treatment data obtained included the reason for not carrying out a cancer-directed operation, radiation treatment, and chemotherapy treatment. Survival data included the survival month and the vital status. Staging of soft tissue sarcoma is typically based on the American Joint Committee for Cancer tumor, nodes, and metastases (AJCC TNM) staging system, in which a localized disease is considered stage I or II, a nodal spread or grade 3 histology is considered stage III, and distant disease is considered stage IV (16). Given the difference in the staging system used in SEER over the years, the historic stage was used in classifying sarcoma into localized disease, regional disease, and/or distant disease.

### Outcome Variables

The primary outcome of this study is 5-year overall survival.

Secondary outcomes of the study include 3-year overall survival, 5-year cancer-specific survival, description of patient demographics and tumor characteristics, demonstrated change of disease incidence over time, and identified factors associated with 5-year overall survival.

### Statistical Analysis

Categorical data were presented with numbers (percentages). Continuous data were presented with median (interquartile range [IQR] or range). The Fisher's Exact test was used to compare categorical variables, and the Kruskal Wallis test was used to compare continuous variables. The age-adjusted incidence rate and the *P*-value were obtained using SEERStat (version 8.3.6). Only the data from 9 registries were used for the evaluation of incidence rate because these were the only registries with data starting from 1975. The Kaplan-Meier curve was used to calculate 5-year survival and the log-rank test to compare crude 5-year survival between different primary sites. The Cox proportional hazard model was used to analyze factors associated with survival. Age and diagnostic year were converted to categorical variables by grouping by decade and SES index, which were then grouped into quartiles in the Cox model to consider non-linear effects. Missing data were imputed with multiple imputations by chained equation. The propensity score was estimated using a logistic regression model based on patient and tumor characteristics for the following kinds of patients: (1) patients who underwent operation and patients who were recommended operation, but the operation was not known to have been performed and (2) patients who received operation only and patients who received operation and additional radiation and/or chemotherapy. Inverse probability treatment weights (IPTW) adjustment was used in the Cox hazard ratio model to examine the treatment effect on survival, adjusting for year of diagnosis, age at diagnosis, race and ethnicity, gender, SES index, tumor grade, stage, laterality, histology, primary site, and tumor size. Statistics were performed by our co-author statistician. The statistical analyses were performed using RStudio (version 1.2.5001).

## Results

### Patient Demographics

**Figure 1:**
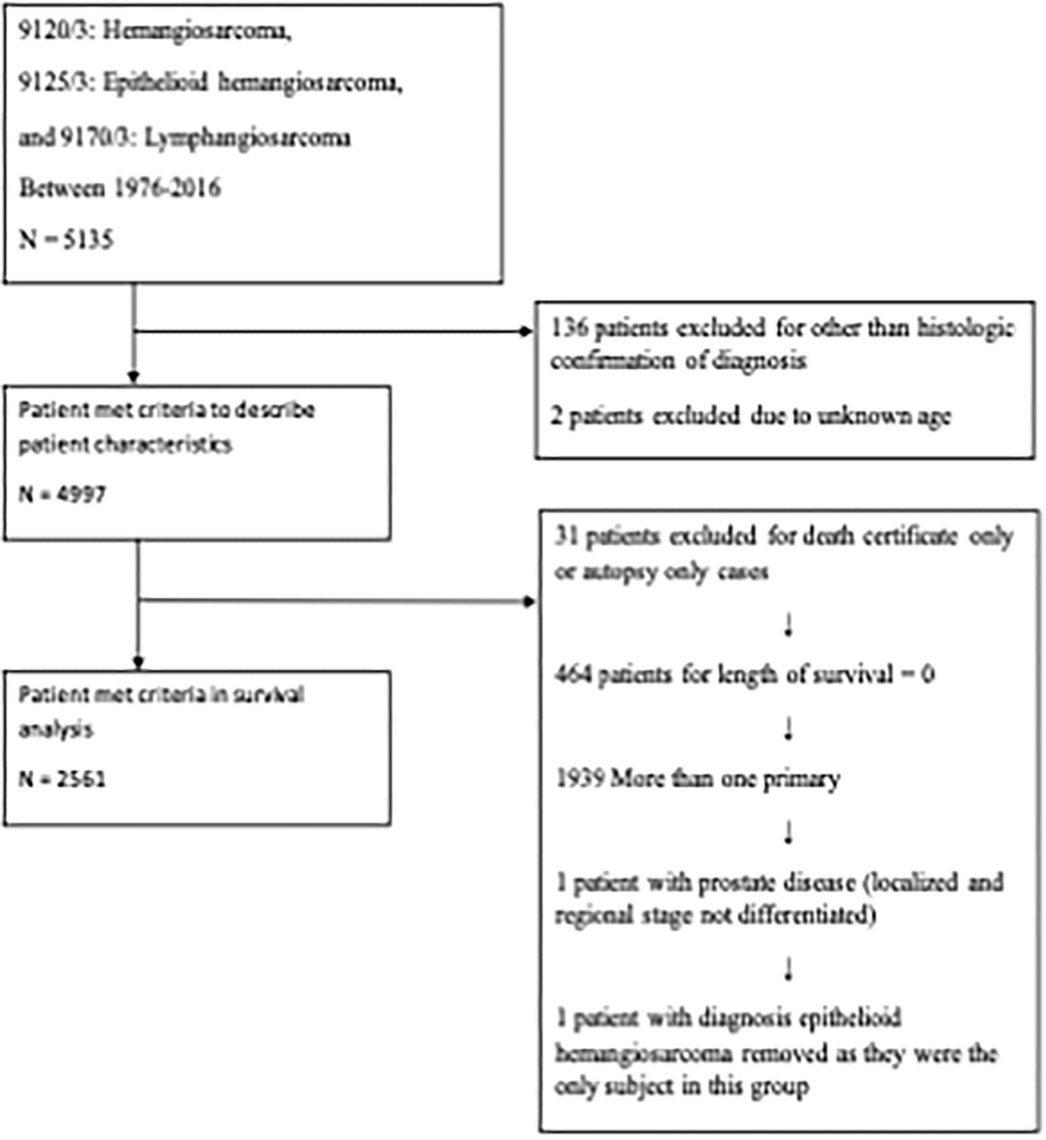
Flow diagram. The Surveillance, Epidemiology, and End Results (SEER) Program 18 Regs Custom Data (with additional treatment fields) was used. All cases from 1976 to 2016 were included.

A total of 5,135 patients with angiosarcoma were identified in the SEER database. Among them, 4,997 patients met the inclusion criteria for all angiosarcoma cohorts, and 2,561 patients met the inclusion criteria for survival analysis (Table 1 and Figure 1). Patients with angiosarcoma were mostly in their 6th decade of life (median age, 69, range, 0–102), were non-Hispanic Caucasian (75.4%), and were more likely to be women (women vs. men, 54 vs. 46%). The database did not provide details on comorbidities. Angiosarcoma of different primary sites had distinct patient characteristics (Table 2). Patients with head and neck tumors were older, that is, they were in the 7th decade of age rather than the 6th decade when patients had tumors in other locations. The predominance of this disease among men was seen in head and neck (69% men), bone (68% men), and visceral (61% men) tumors, while breast angiosarcoma was almost exclusively observed among women (.4% men).

**Table 1:** Patient demographics, tumor characteristics, treatments for all patients with angiosarcoma vs. survival analysis cohort in the Surveillance, Epidemiology, and End Results (SEER) database, 1975–2016.

**Variable, number (%), or median (IQR)**	**Overall cohort**	**^d^Survival analysis cohort**
*n*	4,997	2,561
Year of diagnosis	2007 (2001, 2012)	2006 (1999, 2012)
Age at diagnosis, years	69 (56, 79)	65 (50, 76)
Race and ethnicity		
Non-Hispanic Caucasian	3,770 (75.4%)	1,819 (71%)
Hispanic (All Races)	445 (8.9%)	273 (10.7%)
Non-Hispanic Asian or Pacific Islander	358 (7.2%)	228 (8.9%)
Non-Hispanic African American	379 (7.6%)	211 (8.2%)
Non-Hispanic unknown race	27 (0.5%)	21 (0.8%)
Non-Hispanic American Indian/Alaska native	18 (0.4%)	9 (0.4%)
Gender, male	2,297 (46%)	1,364 (53.3%)
Grade		
Well-differentiated; Grade I	261 (5.2%)	130 (5.1%)
Moderately differentiated; Grade II	402 (8%)	207 (8.1%)
Poorly differentiated; Grade III	859 (17.2%)	445 (17.4%)
Undifferentiated; anaplastic; Grade IV	947 (19%)	460 (18.0%)
Unknown	2,528 (50.6%)	1,320 (51.5%)
Laterality		
Bilateral	26 (0.5%)	12 (0.5%)
No laterality	2511 (50.3%)	1432 (55.9%)
Unilateral	2405 (48.1%)	1082 (42.2%)
Unknown	55 (1.1%)	35 (1.4%)
^a^Primary site		
Soft tissue	1,782 (35.7%)	816 (31.9%)
Head and neck	1,289 (25.8%)	797 (31.1%)
Visceral	865 (17.3%)	479 (18.7%)
Breast	672 (13.4%)	231 (9.0%)
Bone	150 (3%)	98 (3.8%)
Other	118 (2.4%)	78 (3.0%)
Unknown	121 (2.4%)	62 (2.4%)
SES Index	11,284 (10,845, 11,597)	11,261 (10,853-11,588)
^b^Historic stage		
Localized	1,928 (41.1%)	986 (40.8%)
Localized/regional (Prostate cases)	3 (0.1%)	NA
Regional	1,156 (24.7%)	546 (22.6%)
Distant	919 (19.6%)	530 (21.9%)
Unstaged	683 (14.6%)	356 (14.7%)
Tumor size, cm	4.7 (2.4, 8.1)	5 (2.7–9)
^c^Single primary only	2,948 (59%)	NA
Treatment		
Radiation only	212 (4.2%)	141 (5.5%)
Chemotherapy only	438 (8.8%)	291 (11.4%)
Operation only	1,863 (37.3%)	786 (30.7%)
Radiation and chemotherapy	189 (3.8%)	143 (5.6%)
Operation and radiation	667 (13.3%)	424 (16.6%)
Operation and chemotherapy	491 (9.8%)	272 (10.6%)
Operation, radiation, and chemotherapy	286 (5.7%)	208 (8.1%)
No treatment	851 (17.0%)	296 (11.6%)

*IQR, interquartile range; SEER, Surveillance, Epidemiology, and End Results; SES, socioeconomic status*.

a *The soft tissues of the head and neck were grouped into head and neck, the soft tissue of other locations was grouped as soft tissue*.

b *Historic stage is a simplified version of the stage in the SEER database. Prostate cases were excluded from the survival analysis cohort*.

c *Single primary only corresponds to patients whose angiosarcoma is the only primary tumor recorded in the SEER database. Metastatic disease from angiosarcoma is not considered as another primary tumor*.

d *Patients with another primary cancer either prior to or after having an angiosarcoma (including secondary angiosarcomas), death certificate or autopsy cases, and tumors with prostate as the primary site were excluded from survival analysis*.

**Table 2:** Comparison of patient demographics, tumor characteristics, treatment by tumor primary site, all angiosarcoma in SEER database, 1975–2016.

**Variable, N (%) or median (IQR)**	**Bone**	**Breast**	**^a^Head & neck**	**Other**	**^a^Soft tissue**	**Unknown**	**Visceral**	***P*-value**
*n*	150	672	1,289	118	1,782	121	865	
Year of diagnosis	2007 (1996, 2011)	2006 (2000, 2011)	2007 (2000, 2012)	2005 (2000.3, 2011)	2007 (2001, 2012)	2009 (2002, 2013)	2007 (2000, 2012)	0.02
Age at diagnosis	65 (49, 75)	68 (52, 79)	76 (66, 83)	64 (51, 70.8)	68 (55, 78)	66 (54, 75)	62 (48, 73)	<0.001
Race and ethnicity								<0.001
Non-Hispanic Caucasian	116 (77.3%)	525 (78.1%)	1,015 (78.7%)	89 (75.4%)	1,370 (76.9%)	83 (68.6%)	572 (66.1%)	
Hispanic (All Races)	16 (10.7%)	57 (8.5%)	74 (5.7%)	15 (12.7%)	165 (9.3%)	14 (11.6%)	104 (12.0%)	
Non-Hispanic Asian or Pacific Islander	3 (2.0%)	44 (6.5%)	115 (8.9%)	8 (6.8%)	86 (4.8%)	12 (9.9%)	90 (10.4%)	
Non-Hispanic African American	14 (9.3%)	43 (6.4%)	67 (5.2%)	6 (5.1%)	147 (8.2%)	11 (9.1%)	91 (10.5%)	
Non-Hispanic Unknown Race	0	2 (0.3%)	17 (1.3%)	0 (0.0%)	6 (0.3%)	1 (0.8%)	1 (0.1%)	
Non-Hispanic American Indian/Alaska Native	1 (0.7%)	1 (0.1%)	1 (0.1%)	0 (0.0%)	8 (0.4%)	0 (0.0%)	7 (0.8%)	
Gender, male	102 (68.0%)	3 (0.4%)	885 (68.7%)	53 (44.9%)	651 (36.5%)	72 (59.5%)	531 (61.4%)	<0.001
Grade								<0.001
Well-differentiated; Grade I	9 (6.0%)	75 (11.2%)	63 (4.9%)	2 (1.7%)	100 (5.6%)	0 (0.0%)	12 (1.4%)	
Moderately differentiated; Grade II	15 (10.0%)	106 (15.8%)	100 (7.8%)	7 (5.9%)	146 (8.2%)	0 (0.0%)	28 (3.2%)	
Poorly differentiated; Grade III	28 (18.7%)	152 (22.6%)	210 (16.3%)	12 (10.2%)	316 (17.7%)	10 (8.3%)	131 (15.1%)	
Undifferentiated; anaplastic; Grade IV	28 (18.7%)	161 (24%)	180 (14%)	29 (24.6%)	386 (21.7%)	11 (9.1%)	152 (17.6%)	
Unknown	70 (46.7%)	178 (26.5%)	736 (57.1%)	68 (57.6%)	834 (46.8%)	100 (82.6%)	542 (62.7%)	
Laterality								<0.001
Bilateral	1 (0.7%)	2 (0.3%)	9 (0.7%)	0	2 (0.1%)	0	12 (1.4%)	
No laterality	47 (31.3%)	0 (0.0%)	824 (63.9%)	112 (94.9%)	757 (42.5%)	121 (100%)	650 (75.1%)	
Unilateral	99 (66.0%)	670 (99.7%)	427 (33.1%)	6 (5.1%)	1007 (56.5%)	0	196 (22.7%)	
Unknown	3 (2.0%)	0 (0.0%)	29 (2.2%)	0	16 (0.9%)	0	7 (0.8%)	
SES Index	11,207 (10,737, 11,602)	11,339 (10,892, 11,602)	11,263 (10,815, 11,589)	11,342 (10,981, 11,582)	11,317 (10,872, 11,601)	11,206 (10,806, 11,550)	11,225 (10,820, 11,569)	0.4
^b^Historic stage								<0.001
Localized	39 (26.9%)	351 (54.9%)	606 (49.9%)	2 (1.8%)	743 (44.7%)	0 (0.0%)	187 (23.3%)	
Localized/regional (Prostate cases)	0 (0.0%)	0 (0.0%)	0 (0.0%)	0 (0.0%)	0 (0.0%)	0 (0.0%)	3 (0.003%)	
Regional	28 (19.3%)	213 (3.3%)	317 (26.1%)	1 (0.9%)	394 (23.7%)	0 (0.0%)	203 (25.3%)	
Distant	58 (40.0%)	38 (5.9%)	125 (10.3%)	2 (1.8%)	352 (21.2%)	0 (0.0%)	344 (42.8%)	
Unstaged	20 (13.8%)	37 (5.8%)	166 (13.7%)	106 (95.5%)	175 (10.5%)	113 (100.0%)	66 (8.2%)	
Tumor size, mm	58 (37, 82)	40 (20, 74)	34 (18, 60%)	67.5 (32.3, 96.5)	50 (25, 90)	NA	70 (40.5, 100)	<0.001
^c^Single primary	114 (76%)	238 (35.4%)	843 (65.4%)	95 (80.5%)	895 (50.2%)	90 (74.4%)	673 (77.8%)	
Treatment								<0.001
Radiation only	18 (12.0%)	2 (0.3%)	91 (7.1%)	3 (2.5%)	64 (3.6%)	15 (12.4%)	19 (2.2%)	
Chemotherapy only	9 (6.0%)	8 (1.2%)	84 (6.5%)	9 (7.6%)	159 (8.9%)	22 (18.2%)	147 (17%)	
Operation only	43 (28.7%)	392 (58.3%)	394 (30.6%)	55 (46.6%)	777 (43.6%)	3 (2.5%)	199 (23%)	
Radiation and chemotherapy	9 (6.0%)	0 (0.0%)	82 (6.4%)	0 (0.0%)	59 (3.3%)	5 (4.1%)	34 (3.9%)	
Operation and radiation	26 (17.3%)	73 (10.9%)	331 (25.7%)	6 (5.1%)	197 (11.1%)	1 (0.8%)	33 (3.8%)	
Operation and chemotherapy	8 (5.3%)	104 (15.5%)	70 (5.4%)	32 (27.1%)	175 (9.8%)	2 (1.7%)	100 (11.6%)	
Operation, radiation, and chemotherapy	9 (6.0%)	53 (7.9%)	106 (8.2%)	3 (2.5%)	87 (4.9%)	1 (0.8%)	27 (3.1%)	
No treatment	28 (18.7%)	40 (6%)	131 (10.2%)	10 (8.5%)	264 (14.8%)	72 (59.5%)	306 (35.4%)	
5%-yr survival, %	17.8 (12.2–25.8)	44.0 (40.2–48.3)	26.2 (23.7–29.0)	10.2 (5.6–18.6)	32.5 (30.3–34.9)	5.7 (2.5–12.8)	7.8 (6.0–10.1)	<0.001
Cancer-specific survival^d^								<0.001
1-yr	53.4 (45.2–63.1)	95.1 (93.3–96.8)	80.2 (77.9–82.6)	44.2 (35.4–55.3)	75.0 (72.9–77.2)	28.9 (20.0–41.9)	35.9 (32.4–39.8)	
2-yr	44.0 (35.7–54.2)	88.7 (86.0–91.5)	67.0 (64.1–70.0)	31.6 (23.3–43.0)	68.0 (65.6–70.4)	NA	24.1 (20.8–27.8)	
3-yr	41.5 (33.2–52.0)	84.7 (81.5–88.0)	58.8 (55.7–62.1)	NA	65.1 (62.6–67.7)	NA	19.5 (16.4–23.2)	
4-yr	38.2 (28.0–47.7)	81.5 (77.9–85.2)	53.0 (49.7–56.5)	NA	63.8 (61.3–66.5)	NA	17.7 (14.7–21.4)	
5-yr	34.8 (26.2–46.2)	78.8 (74.9–82.9)	48.9 (45.5–52.4)	NA	62.1 (59.5–64.9)	NA	16.3 (13.2–19.5)	

*IQR, interquartile range; SEER, Surveillance, Epidemiology, and End Results; SES, socioeconomic status*.

a *The soft tissues of the head and neck were grouped into head and neck, the soft tissue of other locations was grouped as soft tissue*.

b *Historic stage is a simplified version of the stage in the SEER database*.

c *Single primary only corresponds to patients whose angiosarcoma is the only primary tumor recorded in the SEER database. Metastatic disease from angiosarcoma is not considered as another primary tumor*.

d *Survival rate could not be calculated due to low number s*.

### Tumor Characteristics

Average tumor size was 4.7 cm (range, 1–98.9) at diagnosis. Tumor grades were either high or undifferentiated at presentation according to the available information (grade III, 17.2%, grade IV, 19%, and unknown, 50.6%), but half of the cases were considered localized tumors (Table 1). The most common sites of angiosarcoma were soft tissue (35.7%), followed by head and neck (25.8%), visceral (17.3%), breast (13.4%), and bone (3%). Head and neck tumors tend to be smaller (median 3.4 cm) while visceral angiosarcomas were larger (7 cm) (Table 2). Finally, 41% of patients had another primary tumor of different histology.

### Incidence

**Figure 2:**
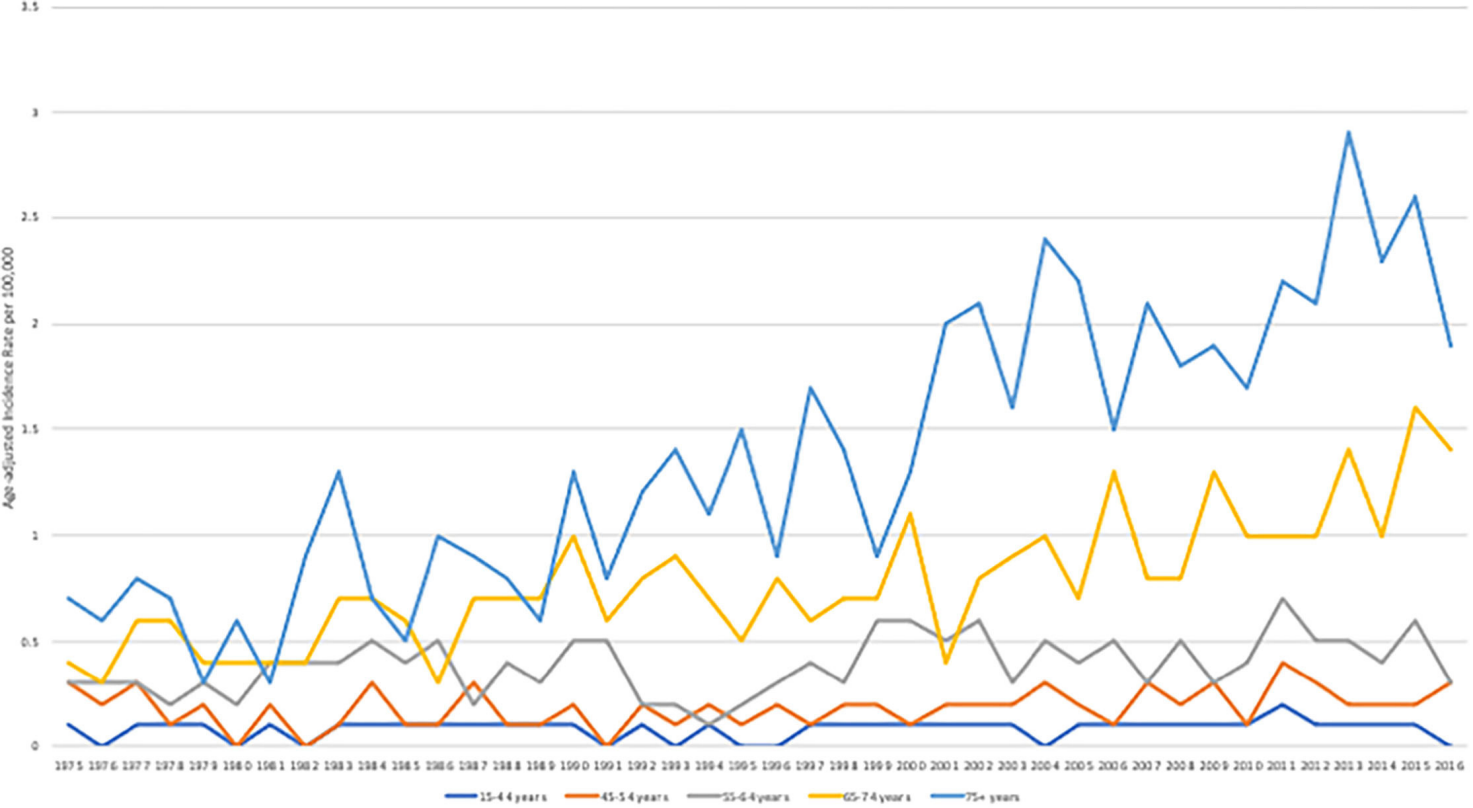
Age-adjusted incidence rate per 100,000 stratified by age groups between 1975 and 2016. The SEER Program 9 Regs Research Data was used. All cases from 9 registries from 1975 to 2016 were included.

Age-adjusted incidence rate of angiosarcoma was 2 per 100,000 population from 1975 to 2016. A slight increase in incidence rate was seen more significantly in older patient groups (65–74 years of age and 75 or older), with a statistically significant higher incidence rate for years 2013, 2015, and 2016 for the age group 65–74 and for years 2011 and 2013–2015 for the age group 75 or older compared to the incidence rate from 1975 to 2016 (*P* < 0.02), Figure 2. Incidence increased from 1 to 3 per 100,000 population from 1975 to 2016, with a higher statistically significant rate in year 2011–2015 (*P* < 0.03).

### Interventions and Outcomes

A total of 4,146 (82.9%) patients underwent intervention. Most patients underwent an operation (66.1%), among which 37.3% underwent operation alone. The remaining portion of patients underwent the following combination therapy: operation with radiation therapy (13.3%), operation with chemotherapy (9.8%), and all three treatments (5.7%) (Table 1). Median overall survival was 13 months (range, 0–483 months) and 3-year and 5-year overall survival were 34.2% (95% CI 32.8–35.6%) and 26.7% (95% CI 25.4–28.1%), respectively. Patients with the primary site of bone were more likely to receive radiation alone or combination treatment. On the other hand, breast tumors were more likely to receive an operation alone while head and neck tumors were more likely to receive radiation and operation. Finally, visceral and other tumor sites were more likely to receive chemotherapy alone or chemotherapy in combination with other therapies (Table 2). The distribution of treatment modality used over time was statistically significantly different (*P* < 0.001). There was an increasing trend of multimodal therapy over surgery alone.

### Factors Associated With 5-Year Overall Survival

**Figure 3:**
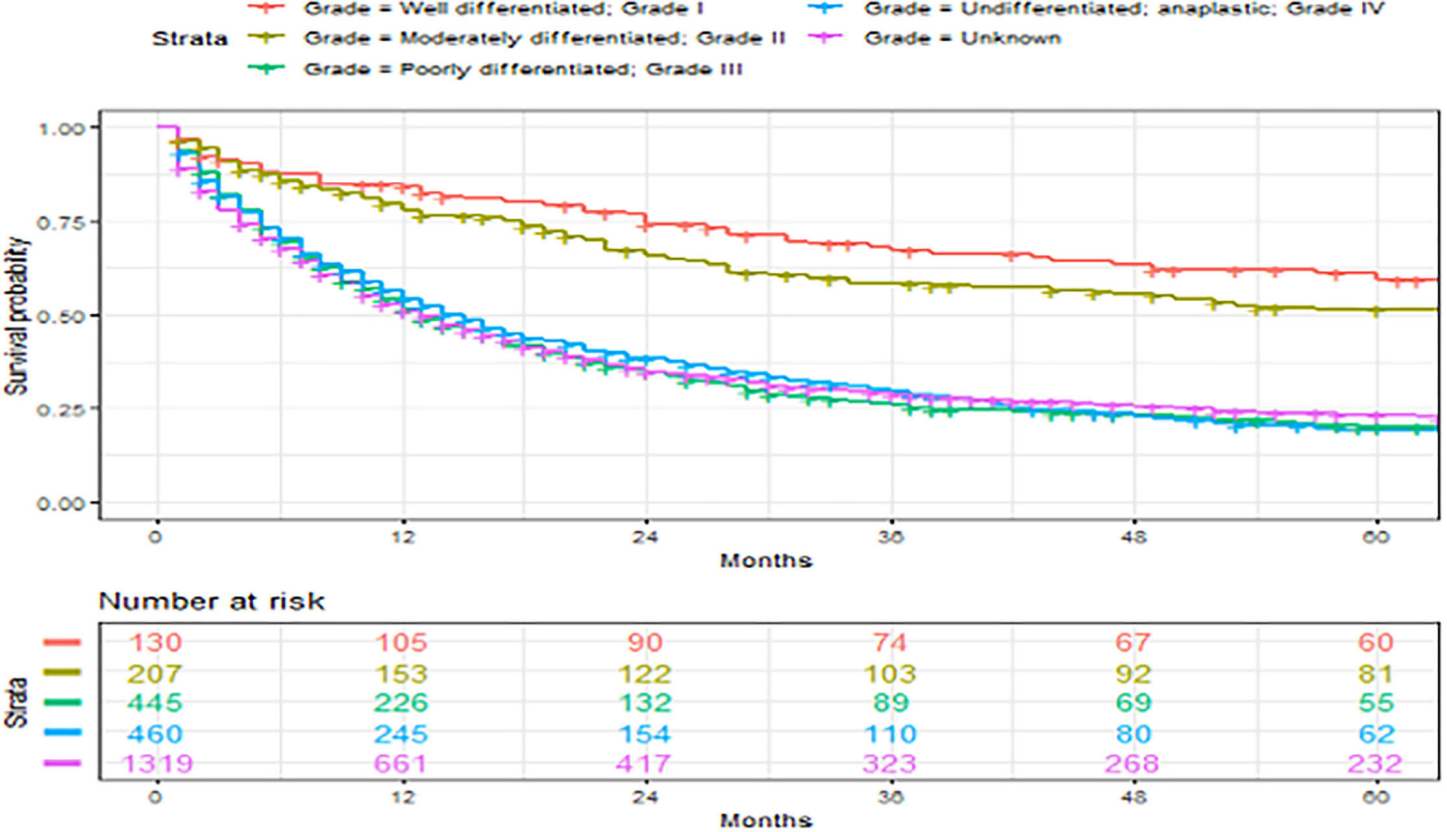
Overall survival of patients with angiosarcoma stratified by primary sites. Survival was calculated using the Kaplan-Meier methods.

**Figure 4:**
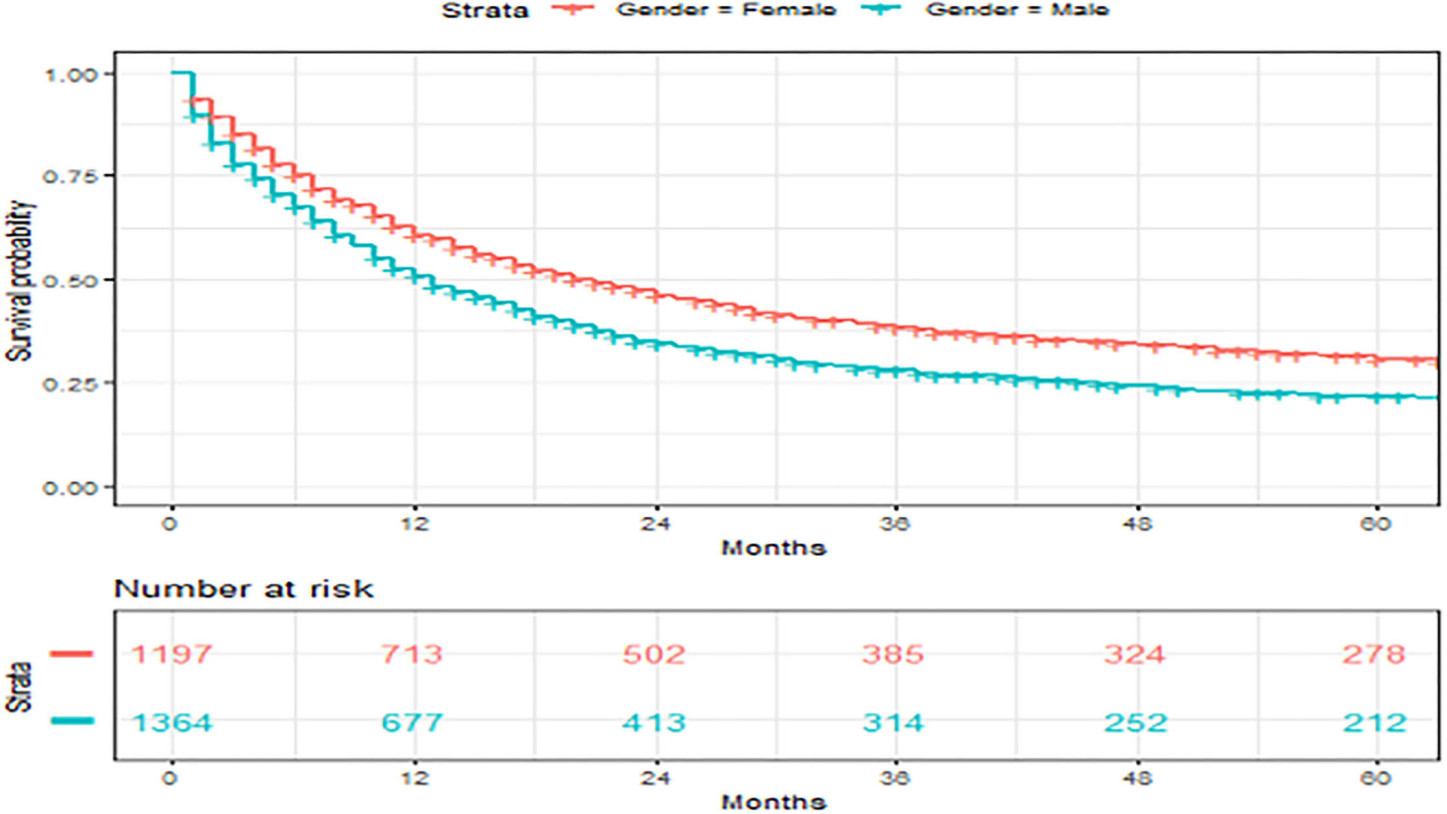
Overall survival of patients with angiosarcoma stratified by gender. Survival was calculated using the Kaplan-Meier methods.

**Figure 5:**
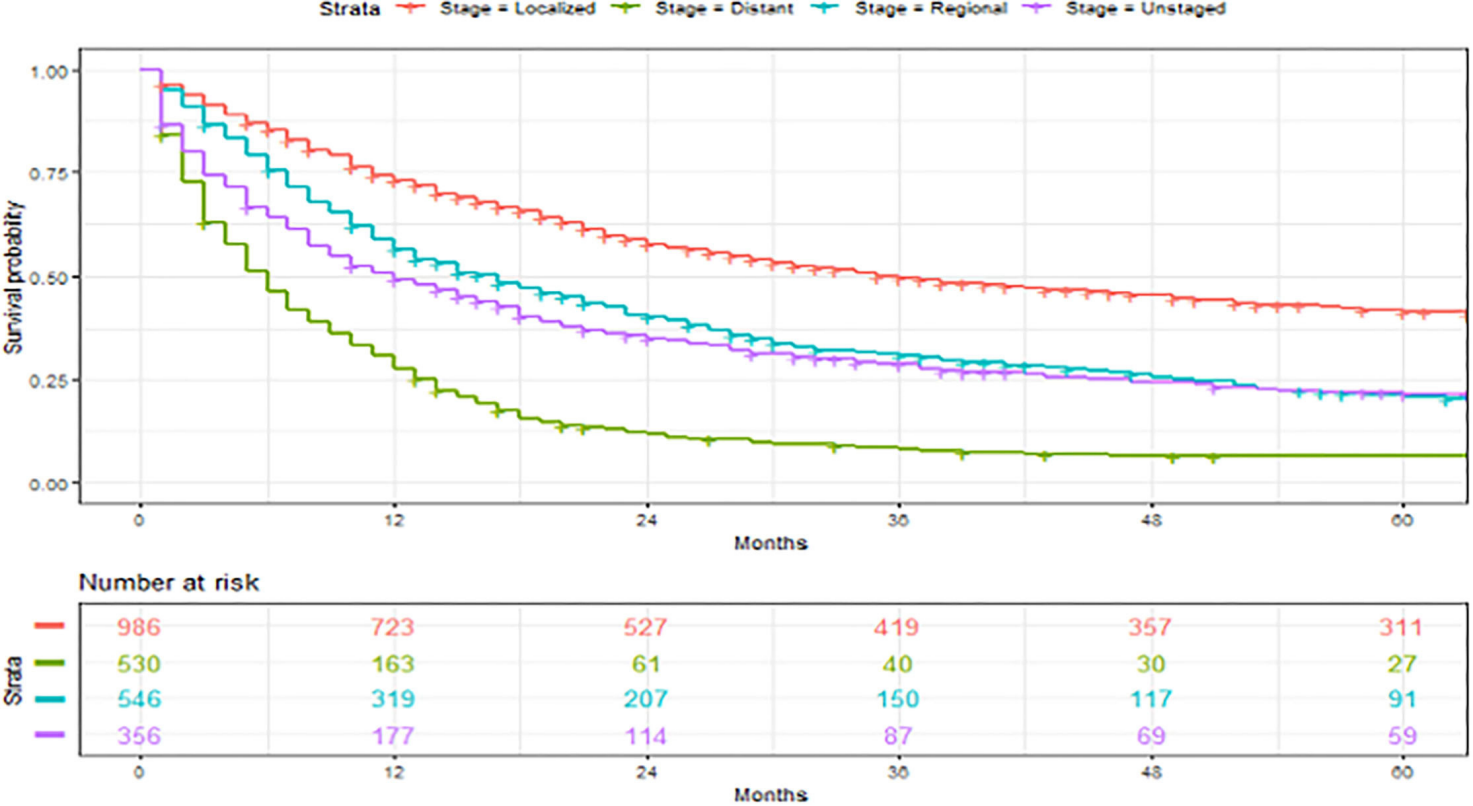
Overall survival of patients with angiosarcoma stratified by historic stage. Survival was calculated using the Kaplan-Meier methods.

**Figure 6:**
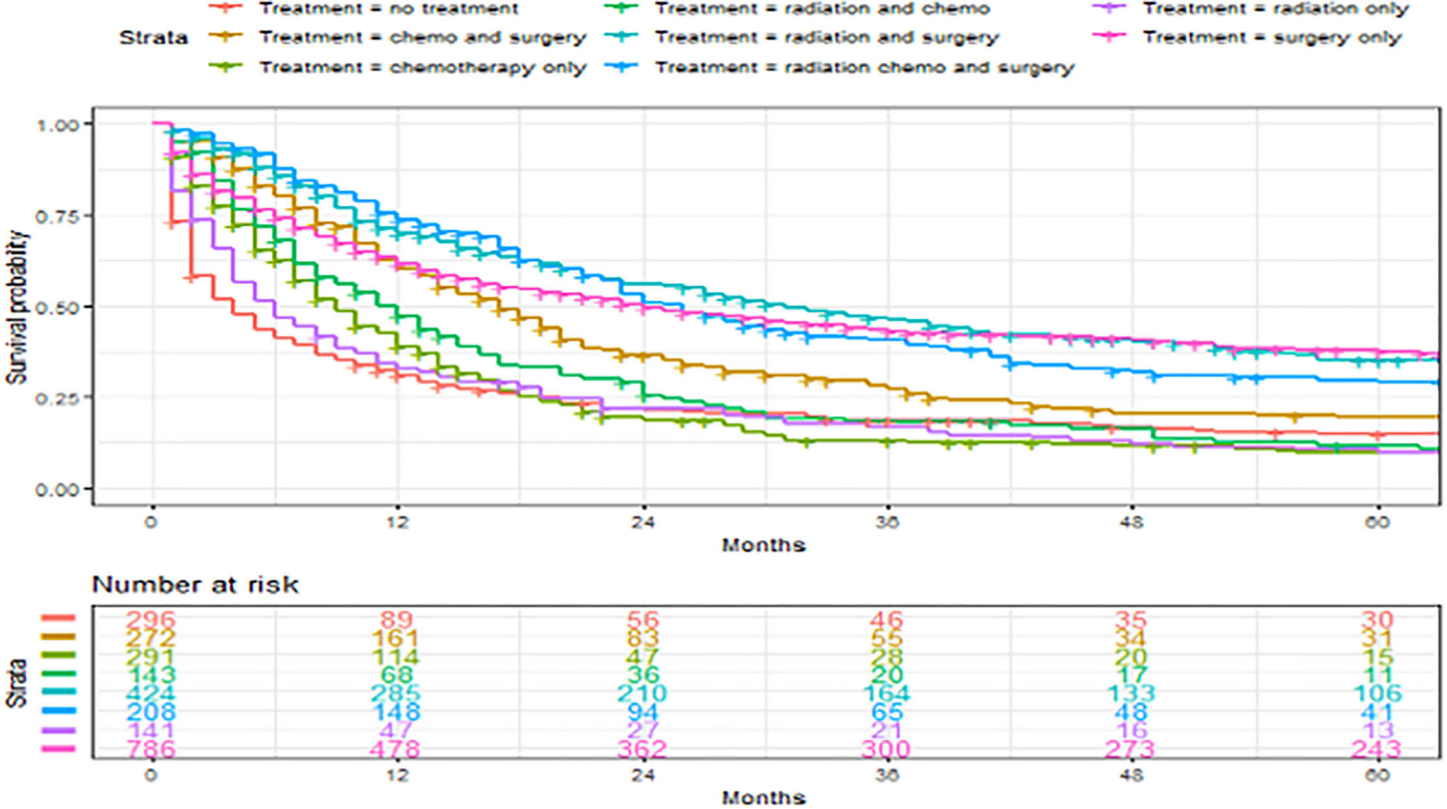
Overall survival of patients with angiosarcoma stratified by treatment. Survival was calculated using the Kaplan-Meier methods.

A total of 2,561 patients with primary angiosarcoma met the criteria for survival analysis (Table 1). Men (hazard ratio [HR] 1.17, 95% CI 1.06–1.29), older age (10-year increments) (1.28, 1.24–1.32), higher tumor grade (reference = grade I; grade II 1.4, 1–1.91; grade III 2.4, 1.88–3.21; grade IV 2.53, 1.88–3.37), advanced stage (reference = localized; regional 1.5, 1.33–1.7; distant 2.91, 2.53–3.33), tumor without laterality (reference = unilateral; 1.31, 1.18–1.46), primary site of visceral (reference = soft tissue; 1.73, 1.51–1.98), and larger tumor size (1-cm increments) (1.02, 1.01–1.02) were associated with poor outcomes according to the multivariate analysis (Table 3, Figures 3–6). Female and male patients differed in age, tumor differentiation, tumor location, and treatment patterns (Table 4). After adjusting for variables, men were still found to be associated with an increased risk of mortality of 17%. Any single or combined treatments, except for radiation therapy alone (HR.85, 95% CI.68–1.06), and primary tumor sites of head and neck (reference = soft tissue; 8, 71–0.91) were associated with improved outcomes. In the decade when angiosarcoma was diagnosed, SES index, race, and ethnicity were not associated with mortality. IPTW with the adjusted Cox proportional hazard model demonstrated worse survival if the patient underwent an operation after recommendation compared to those who did not (HR 1.2, 95% CI 1.04–1.39, *P* < 0.001) before month 25. Patients who underwent an operation after recommendation were found to have better survival (HR.67, 95% CI.52-0.88, *P* < 0.001) after month 25. Additionally, patients who underwent operation alone were associated with poor survival compared to those who underwent operation with either adjuvant or neoadjuvant radiation and/or chemotherapy (HR 1.37, 95% CI 1.17–1.6, *P* < 0.001) before month 25, but they showed better survival (HR.61, 95% CI.46-0.8, *P* < 0.001) after the same month.

**Table 3:** The Cox regression model of patient survival in patients with primary angiosarcoma in SEER database, 1975–2016.

	**HR**	**95%CI**	***P*-value**
Age at diagnosis	1.28	(1.24, 1.32)	<0.001
Gender			
Female (Ref)			
Male	1.17	(1.06, 1.29)	0.001
Year of diagnosis	0.97	(0.93, 1.02)	0.27
SES index	0.95	(0.89, 1.01)	0.10
8,201–9,100 (Ref)			
9,101–10,000	0.95	(0.70, 1.28)	0.72
10,000–10,900	0.82	(0.62, 1.08)	0.17
10,900–11,800	0.83	(0.64, 1.07)	0.16
Grade			
Well-differentiated; Grade I (Ref)			
Moderately differentiated; Grade II	1.40	(1.00, 1.91)	0.05
Poorly differentiated; Grade III	2.40	(1.79, 3.21)	<0.001
Undifferentiated; anaplastic; Grade IV	2.52	(1.88, 3.37)	<0.001
Unknown	2.12	(1.60, 2.80)	<0.001
Histology			
Hemangiosarcoma (Ref)			
Lymphangiosarcoma	0.76	(0.44, 1.33)	0.34
^a^ History stage			
Localized (Ref)			
Regional	1.50	(1.33, 1.70)	<0.001
Distant	2.91	(2.53, 3.33)	<0.001
Unstaged	1.28	(1.07, 1.53)	0.009
Laterality			
Unilateral (Ref)			
Unknown	0.95	(0.65, 1.39)	0.79
Bilateral	1.17	(0.62, 2.21)	0.62
No laterality	1.31	(1.18, 1.46)	<0.001
^b^Primary site			
Soft tissue (Ref)			
Head& neck	0.80	(0.71, 0.91)	<0.001
Breast	0.92	(0.74, 1.15)	0.47
Bone	1.26	(0.98, 1.64)	0.07
Visceral	1.73	(1.51, 1.98)	<0.001
Other	1.95	(1.44, 2.63)	<0.001
Unknown	2.20	(1.59, 3.05)	<0.001
Race and origin			
Hispanic (All Races) (Ref)			
Non-Hispanic Unknown Race	0.20	(0.09, 0.45)	<0.001
Non-Hispanic African American	0.84	(0.68, 1.04)	0.10
Non-Hispanic Caucasian	0.90	(0.77, 1.05)	0.16
Non-Hispanic Asian or Pacific Islander	0.98	(0.80, 1.20)	0.82
Non-Hispanic American Indian/Alaska Native	1.12	(0.49, 2.56)	0.78
Tumor size (cm)	1.02	(1.01, 1.02)	<0.001
Treatment			
No treatment (Ref)			
Operation	0.56	(0.47, 0.66)	<0.001
Radiation	0.85	(0.68, 1.06)	0.15
Chemotherapy	0.63	(0.52, 0.76)	<0.001
Operation and radiation	0.45	(0.38, 0.55)	<0.001
Operation and chemotherapy	0.50	(0.41, 0.62)	<0.001
Radiation and chemotherapy	0.58	(0.46, 0.74)	<0.001
Operation, radiation, and chemotherapy	0.48	(0.38, 0.60)	<0.001

*SEER, Surveillance Epidemiology, and End Results; SES, socioeconomic status*.

a *Historic stage is a simplified version of the stage in the SEER database*.

b *The soft tissues of the head and neck were grouped into head and neck, the soft tissue of other locations was grouped as soft tissue*.

**Table 4:** Comparison of women vs. men in patients with primary angiosarcoma in SEER database, 1975–2016.

**Variable, Number (%), or Median (IQR)**	**Female**	**Male**,	***P*-value**
*n*	1,197	1,364	
Year of diagnosis	2005 (1998, 2011)	2007 (2000, 2012)	<0.001
Age at diagnosis	63 (47, 76)	67 (53, 76.3)	0.001
Race and ethnicity			0.26
Non-Hispanic Caucasian	829 (69.3%)	990 (72.6%)	
Hispanic (All Races)	138 (11.5%)	135 (9.9%)	
Non-Hispanic Asian or Pacific Islander	103 (8.6%)	125 (9.2%)	
Non-Hispanic African American	112 (9.4%)	99 (7.3%)	
Non-Hispanic Unknown Race	11 (0.9%)	10 (0.7%)	
Non-Hispanic American Indian/Alaska Native	4 (0.3%)	5 (0.4%)	
Grade			<0.001
Well differentiated; Grade I	86 (7.2%)	44 (3.2%)	
Moderately differentiated; Grade II	127(10.6%)	80 (5.9%)	
Poorly differentiated; Grade III	213 (17.8%)	232 (17%)	
Undifferentiated; anaplastic; Grade IV	217 (18.1%)	243 (17.8%)	
Unknown	554 (46.3%)	765 (56.1%)	
Laterality			<0.001
Bilateral	3 (0.3%)	9 (0.7%)	
No laterality	615 (51.4%)	817 (59.9%)	
Unilateral	565 (47.2%)	517 (37.9%)	
Unknown	14 (1.2%)	21 (1.5%)	
^a^Primary site			<0.001
Head and neck soft tissue	297 (24.8%)	519 (38.0%)	
Soft tissue (other than head and neck)	380 (31.7%)	417 (30.6%)	
Visceral	192 (16.0%)	287 (21.0%)	
Breast	230 (19.2%)	1 (0.1)	
Bone	32 (2.7%)	66 (4.8%)	
Other	42 (3.5%)	36 (2.6%)	
Unknown	24 (2%)	38 (2.8%)	
SES index	11259 (10,820, 11,594)	11263 (10,864.5, 11,586.5)	0.75
^b^Historic stage			<0.001
Localized	524 (45.8%)	462 (36.2%)	
Regional	237 (20.7%)	309 (24.2%)	
Distant	209 (18.3%)	321 (25.2%)	
Unstaged	173 (15.1%)	183 (14.4%)	
Tumor size, cm	5 (2.9, 9)	5.2 (2.6, 9)	0.59
Treatment			<0.001
No treatment	119 (9.9%)	177 (13%)	
Radiation only	57 (4.8%)	84 (6.2%)	
Chemotherapy only	118 (9.9%)	173 (12.7%)	
Operation only	433 (36.2%)	353 (25.9%)	
Radiation and chemotherapy	50 (4.2%)	93 (6.8%)	
Operation and radiation	199 (16.6%)	225 (16.5%)	
Operation and chemotherapy	126 (10.5%)	146 (10.7%)	
Operation, radiation, and chemotherapy	95 (7.9%)	113 (8.3%)	

*SEER, Surveillance, Epidemiology, and End Results; SES, socioeconomic status*.

a *The softs tissue of the head and neck were grouped into head and neck, the soft tissue of other locations was grouped as soft tissue*.

b *Historic stage is a simplified version of the stage in the SEER database*.

## Discussion

Angiosarcoma is a rare but aggressive malignancy with an age-adjusted incidence rate of 1–0.3 per 100,000 population that has risen slightly, especially in the older population. The overall 5-year survival was 27% in our cohort. We found that younger age, female gender, lower tumor grade, localized disease, primary sites of head and neck, and all treatment, except for radiation therapy alone, were associated with improved survival in patients with primary angiosarcoma. Primary and secondary angiosarcomas originated from different primary sites had a distinct patient and tumor characteristics with different survival. Operation and multimodal therapy were associated with improved 5-year survival. This study is novel because it allowed a for a unique examination of much larger cohorts than those currently reported in the literature. This study also used population-based data to describe angiosarcoma outcomes and used inverse probability treatment weights to adjust for confounders to evaluate the survival benefit of the operation and multimodal therapy.

Soft tissues of trunk and extremity (36%) were the most common locations of angiosarcoma, followed by head and neck soft tissues (26%), visceral (17%), and breast (12%) angiosarcomas. In comparison, head and neck tumors were most common in the findings of Wang et al., which was likely due to the differences in the patient population (1). In our cohort, head and neck tumors (30%) were also more common than soft tissues of trunk and extremity (24%) in non-Hispanic Asians or Pacific Islanders. Angiosarcoma of the great vessel is extremely rare, with mostly case reports or small case series in the literature (17–19). A case series that reported 13 patients with primary angiosarcoma of the heart, aorta, and great vessels from 1985 to 2011 has found that the majority of patients had metastatic diseases at the time of presentation and had very poor prognoses (19).

We found that patients with angiosarcoma were mostly in the 6th decade of their life similar to previous studies (3, 8, 20). Patients with head and neck angiosarcomas were older (7th decade of life) (1, 21). Wang et al. found similar trends but patients with visceral and breast angiosarcomas in their cohort were much younger, with a mean age of 46 and 39, respectively, compared to the median age of 62 and 68 in our cohort (1). A meta-analysis on liver angiosarcoma had a median patient age of 58 and a meta-analysis of breast angiosarcoma had a mean patient age of 69 (8, 9). These differences in patient demographics could be due to differences in patient population or Chang et al. could have included more patients with primary breast angiosarcoma which occurred at a younger age (20). We had a slightly higher percentage of women, 54%, similar to other unselected series of angiosarcoma (1–3). The predominance of men who were diagnosed with head and neck angiosarcomas had been reported and confirmed in our cohort (1, 7, 13, 21, 22). Additionally, visceral and bone angiosarcomas had a slight predominance in men (61 and 68%, respectively), while breast angiosarcoma was almost exclusively among women. A meta-analysis of liver angiosarcoma showed its predominance among women, in which only 36% were men (9). This was possibly due to the selection bias of the meta-analysis or due to our inclusion of other viscera.

In our entire cohort, median survival was 13 months with a 5-year overall survival of 27%. Looking at all angiosarcomas, patients with breast angiosarcoma had the best 5-year overall survival of 44, followed by 33% in those with soft tissue angiosarcoma. Patients with visceral tumor had a poor 5-year overall survival of 8%. Wang et al. and Fayette et al. reported 5-year overall survival for unselective angiosarcoma of 43 and 41%, respectively (1, 2). Higher survival in Wang et al. could be explained by their 87.5% 5-year survival for breast angiosarcoma and higher 5-year survival in visceral angiosarcoma of 40.8%. The reason for the difference in survival was likely due to the higher percentage of low-grade tumors seen in patients with visceral and breast angiosarcomas, as well as the younger age of patients with breast angiosarcoma in the study of Wang et al. (1). The survival rates of breast angiosarcoma in other studies were less optimal and closer to our cohort (2, 8). Higher survival in Fayette et al. could be explained by a large percentage of breast angiosarcoma (35%) in their cohort. Additionally, soft tissue angiosarcoma had 74% 5-year survival in the study of Fayette et al. compared to 32.5% in our findings and 40% in the study of Sinnamon et al. (7). The reason for the high survival of patients with soft tissue angiosarcoma in the study of Fayette et al. is unknown. The poor survival in patients with visceral angiosarcoma has been previously reported with results similar to ours (2, 9, 12).

We found that younger age, female gender, lower tumor grade, localized disease, smaller tumor size, and the primary sites of head and neck were independent patient and tumor characteristics that were associated with improved survival. Factors associated with survival were highly varied in different studies in literature due to the diverse selection of patient cohort, difference in the grouping of continuous variables, and various variables assessed (1, 2, 7–11, 13, 20, 21, 23–26). Younger age (60 or 70 as the cutoff), localized tumor, low grade, and small tumor size (5 cm as the cutoff) were frequently associated with better survival (1, 7–11, 13, 21, 23–25). However, these factors were not significantly associated with survival in other studies (2, 8–11, 20, 25). Our study found that younger age, smaller size, low grade, and localized disease were all independently associated with overall survival. This was likely due to our increased power with this larger cohort.

We found improved survival in women, which has not been reported. While there were many differences in baseline characteristics of men vs. women, this significance persisted even after adjusting for other variables. Two previous studies had associated men with improved outcomes in univariate analysis but were both lost in multivariate analysis (13, 21). This suggested that gender differences in the outcome should be further explored. We did not find diagnosis year or SES to be associated with angiosarcoma survival.

Breast angiosarcoma had the best overall survival of 44%, whereas head and neck tumor had a relatively poor survival of 26% in our cohort when both primary and secondary angiosarcomas were included. However, after excluding secondary angiosarcomas and patients with other primary malignancies in the SEER database and adjustment of other covariates, head and neck locations were associated with a 20% decrease in risk of mortality, while breast location was not significantly different compared to soft tissue. Secondary breast angiosarcoma has better prognosis compared to primary breast angiosarcoma, which could explain the high unadjusted survival in unselected breast angiosarcoma (27). Another explanation for the observed poor survival in head and neck tumors and the improved survival in breast tumors is that the survival of patients with angiosarcoma was mostly related to other patient and tumor characteristics, such as grade, stage, and whether amenable to surgical treatment.

Operation is the primary treatment for all angiosarcomas [28]. In our cohort, treatment received differed in patients with different primary sites. Operation alone as primary treatment was more common in breast tumors similar to the review of breast angiosarcoma by Depla et al. (8) Additionally, it was also common in our cohort for patients with soft tissue angiosarcoma to receive operation alone. Chemotherapy alone was relatively more common in visceral angiosarcoma in our cohort, which is likely related to multifocal disease or the advanced stage of the visceral tumor (12). Radiation plays a larger role in head and neck angiosarcoma, with a study of scalp angiosarcoma showing that 72% received radiation mostly in addition to operation (13). This was confirmed in our cohort. A higher percentage of patients with visceral and bone tumors did not receive any treatment in our cohort, which may be related to the severity of the disease. This difference in treatment was not observed in the study of Wang et al. (1).

Evidence on survival benefits of current treatments are poor as they show mixed results. Operation was associated with improved disease-free and overall survival in univariate analysis of primary head and neck angiosarcomas both based on the SEER database and the meta-analysis, but statistical significance was lost in the multivariate analysis used in the study based on SEER data (10, 21). In an osseous angiosarcoma study based on SEER data, operation was associated with improved overall and case-specific survival in both univariate and multivariate analysis (11). In a study on non-metastatic scalp angiosarcoma based on National Cancer Database, operation plus postoperative radiation and/or chemotherapy were associated with improved survival compared to definitive radiation and/or chemotherapy alone (13). However, in other studies, operation was not associated with survival, and in one study, it was even associated with worse survival likely due to the fact that it was unadjusted (9, 25, 26). Wang et al. and Patel et al. demonstrated improved overall survival in patients who underwent multimodal therapy (1, 22). Radiation in addition to operation has been associated with improved recurrence-free survival and overall survival in patients with breast angiosarcoma (20, 23). However, most other studies on angiosarcoma have not found radiation or chemotherapy to be associated with survival (7–11). Wang et al. again found radiation to be negatively associated with survival likely due to confounders (26). In our study, radiation alone was the only treatment modality that was not associated with improved survival in the Cox model. IPTW with the adjusted Cox model found that having operation after provider recommendation and the use of multimodal therapy compared to operation alone was associated with worse survival initially but improved survival after 2 years. This worse short-term survival may be associated with the physiological stress induced by operation, chemotherapy, or radiation. Currently, the only guideline on the treatment of angiosarcoma specifically was published in 2013 by the Dermatologic Cooperative Oncology Groups of the German Cancer Society which recommended operation with adjuvant radiation therapy (6).

### Limitations

This is a retrospective study based on a national registry. The tumor stage was not available for all locations during certain years, and tumor size was not reported past 98.9 cm. Patients with unknown radiation therapy or chemotherapy were grouped together with patients that did not undergo radiation therapy or chemotherapy. Furthermore, we lacked details regarding resection margin, local recurrence, history of lymphedema or prior radiation therapy, specific chemotherapy regimen, immunotherapy information, and the specific cause of death. We evaluated overall survival instead of cancer-specific survival. The tumor stage, grade, and tumor location were extremely heterogeneous in this study. Thus, we chose propensity score matching to determine the survival benefit of the operation and neoadjuvant/adjuvant therapy, which addressed confounding from other variables. Additionally, while we excluded patients with prior cancer for survival analysis, it was possible that some patients with radiation-induced angiosarcoma were still included in the cohort. Despite these limitations, this is by far the largest study that explored the outcomes of angiosarcoma on a national multi-institutional level.

## Conclusions

Angiosarcoma of different primary sites has distinct patient and tumor characteristics as well as survival. Younger age, female gender, lower tumor grade, localized disease, primary site of head and neck, and all treatment options, except for radiation therapy alone, were associated with improved survival. Operation when recommended and the use of multimodal therapy in addition to operation could improve the 5-year survival. Further studies are needed to clarify the reason behind the short-term higher risk of mortality after surgery and multimodal therapy to guide clinical practice and for the development of treatment guidelines.

## Data Availability Statement

The datasets presented in this study can be found in online repositories. The names of the repository/repositories and accession number(s) can be found below: SEER.

## Author Contributions

QY, ME, and MD were responsible for study planning. QY and MD were responsible for data collection. RF, QY, JG, and MD was responsible for statistical analysis. ME, QY, and MD was responsible for manuscript. All authors were responsible data interpretation and drafting responsible for critical revision.

## Conflict of Interest

The authors declare that the research was conducted in the absence of any commercial or financial relationships that could be construed as a potential conflict of interest.

## Publisher's Note

All claims expressed in this article are solely those of the authors and do not necessarily represent those of their affiliated organizations, or those of the publisher, the editors and the reviewers. Any product that may be evaluated in this article, or claim that may be made by its manufacturer, is not guaranteed or endorsed by the publisher.
